# Bacterial Cellulose-Based Superabsorbent Hydrogel for Wet Wound Dressing

**DOI:** 10.3390/molecules30030737

**Published:** 2025-02-06

**Authors:** Meiqing Mo, Chaojun Wu, Yehong Chen

**Affiliations:** State Key Laboratory of Biobased Material and Green Papermaking, Qilu University of Technology (Shandong Academy of Sciences), Jinan 250353, China; meiqingmo123@163.com (M.M.); chenyh@qlu.edu.cn (Y.C.)

**Keywords:** bacterial cellulose, absorbent dressing, moisturizing wound

## Abstract

Absorption of exudates is crucial for moist wound treatment, particularly in chronic wound care applications. However, controlling wound exudates with current gel-based wound dressings has challenges, such as the risk of bacterial infection during long-term transportation and use. In this study, a bacterial cellulose (BC)/polyvinyl alcohol (PVA) composite hydrogel (PBC) was prepared by a simple method using citric acid (CA) as the crosslinking agent. Fourier-transform infrared spectroscopy showed that the reaction between the carboxyl group of CA and the hydroxyl group of the BC-PVA hydrogel enhanced its hydrophilicity. Sol–gel analysis confirmed that an increase in the PVA content led to stronger crosslinking of the polymer network in the hydrogel. Wide-angle X-ray diffraction results showed that at low PVA concentrations, the tendency to connect with cellulose chains and crystallinity increased. In addition, the hydrogel dressing demonstrated excellent water absorption capacity; the swelling rate reached 3485.3% within one hour, and no cytotoxic effect was observed on the L929 fibroblast line in vitro. The designed hydrogel exhibited the ability to resist bacteria. Therefore, the new PBC biomaterial has certain potential for various applications, particularly as a highly absorbent dressing.

## 1. Introduction

When human body tissue is damaged, the integrity of the skin tissue structure is eventually restored when the wound heals. Wound healing is mainly divided into four stages: hemostasis, inflammation, cell proliferation, and maturation and remodeling [1]. However, chronic wounds typically exhibit an extended healing process and often remain in the inflammatory stage, leading to deterioration of the original damage [2]. For patients with certain diseases (such as diabetes), this situation may be serious or even life-threatening [3]. In recent years, simple cloth dressings have been replaced with various functional wound dressings, such as alginate, semi-permeable membranes, bubble membranes, hydrogels, electrospinning materials, hydrophilic colloids, and other nanofiber dressings, providing a suitable environment for wound healing and restoring auxiliary functions [4,5]. These wound dressings provide a moist wound environment, remove wound exudates, and prevent secondary infections [6]. According to a theory introduced by George Winter in 1962, wet wounds heal faster than dry wounds [7]. The beneficial effects of a damp wound environment include the prevention of tissue or cell death due to dehydration and accelerated angiogenesis [8]. When dermal tissue is exposed to air, it becomes dehydrated, and the epidermis migrates under the dehydrated tissue, where it obtains sufficient water to survive. However, when a moist wound dressing is used to keep the surface of a wound moisturized, the epidermis will quickly migrate to the surface of the dermis, which promotes the formation of granulation tissue and re-epithelialization of the treated wound, thereby accelerating wound healing [9,10].

Among all wound dressings, hydrogels have been widely applied owing to their good biocompatibility, water absorption, moisture retention, and permeability [11,12,13]. Unlike gauze, which mainly provides a barrier through the evaporation of wound exudates to keep the wound dry, hydrogel wound dressings can interact with the wound surface to maintain a moist local environment and stimulate healing by providing a series of cells and cytokines required for wound repair [14]. In addition, hydrogels can be peeled off without any damage to the regenerated surface and without leaving any residual dressing material on the wound. Equally importantly, they can lock the exudate in the dressing, thereby minimizing bacterial spread when removed from the wound surface [15]. However, limited fluid absorption limits their use in highly exudative wounds. Therefore, it is necessary to further optimize the liquid absorption of hydrogels by providing porous structures and elastic networks.

In the past two decades, owing to its controllable structures and properties, polyvinyl alcohol (PVA) has been used to develop hydrogel dressings [16]. However, the instability of PVA biopolymers in water and their poor swelling ability limit their applications in the biomedical field [17]. The introduction of the natural polymer bacterial cellulose (BC) into a PVA hydrogel system is expected to overcome these limitations. Bacterial cellulose is a natural nanofiber produced by Komagataeibacter xylinus. BC has high purity, a 3D ultrafine network structure, low immunogenicity, and good interaction with different cell types, making it an attractive material for wound healing and wound care products [18,19]. Studies have been conducted on the application of BC-PVA hydrogels in wound dressings [20,21]. Unfortunately, the swelling and water retention rates of the hydrogels in previous studies were not optimistic, and the swelling rate was only 240%. Various crosslinking agents, such as epichlorohydrin and glutaraldehyde, have been widely used to enhance PVA- and BC-based hydrogels [22,23]. However, these reagents may exert toxic effects, limiting their biomedical applications. Therefore, it is necessary to develop non-toxic and green crosslinking agents for biopolymers. Citric acid (CA) is the main organic acid in citrus fruits and plays a crucial role in metabolism. CA can react with the hydroxyl and amino groups of biopolymers, making it a green crosslinking agent for them [24]. In addition, crosslinking between polymers and CA provides free carboxylic acid groups, which can enhance biocompatibility and regulate the hydrophilicity of polymer surfaces [25]. Therefore, in this study, CA was used as a green crosslinking agent to prepare PVA-BC hydrogels. To the best of our knowledge, there has been no prior research on the development and preparation of BC-PVA hydrogels crosslinked with CA.

This study involves the development and characterization of a BC-PVA-CA hydrogel for moist wound management. The proposed hydrogel was developed with a simple two-step reaction. First, a PVA-BC (PB) hydrogel was synthesized using a simple physical crosslinking freeze–thaw method. It was then immersed in CA and catalyst (CAT) solutions (disodium phosphate and sodium bicarbonate), which significantly improved the swelling, absorption, antibacterial, and water retention properties of the PVA-BC-CA (PBC) hydrogel. These properties of the hydrogel demonstrate its potential as a dressing material. Further, the surface porosity could be controlled by changing the PVA content. The biocompatibility of the hydrogel and L929 was also evaluated to show its utility for biomedical applications.

## 2. Results and Discussion

### 2.1. Hydrogel Synthesis

**Figure 1:**
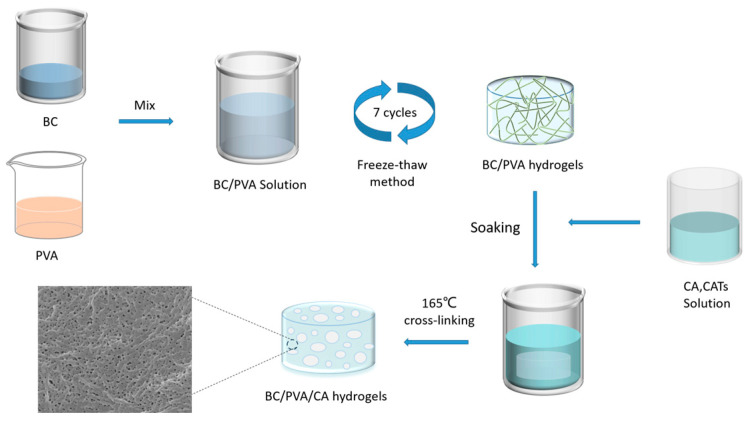
Schematic diagram of hydrogel preparation process.

To prepare the PBC hydrogels, BC was added to a PVA solution to obtain PVA-BC solutions with different PVA concentrations. The hydroxyl groups in the PVA molecular chain can form intermolecular or intramolecular hydrogen bonds. BC contains multiple hydroxyl groups that interact with the hydroxyl groups of PVA to form hydrogen bonds. After freezing and thawing, microcrystalline domains formed PVA-BC hydrogels. Then, the hydrogels were heat-crosslinked with CA and CAT at 165 °C to promote the esterification reaction between CA and the hydrogel hydroxyl groups, which greatly improved the water absorption performance of the hydrogels. Each hydrogel was freeze-dried to form a frozen gel. Figure 1 depicts the preparation process in detail.

### 2.2. FT-IR Characterization Analysis of the Hydrogels

**Figure 2:**
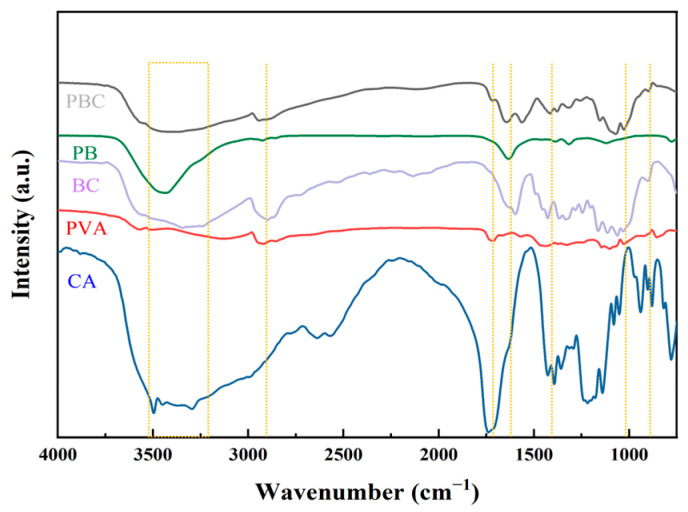
FT-IR spectra of BC, PVA, CA, PB, and PBC4 hydrogel.

The composition and structural changes of the hydrogels and pure polymers (BC, CA, and PVA) were analyzed in the wavelength range of 400–4000 cm^−1^ using FT-IR (Figure 2). All the prepared hydrogels showed wide and strong bands at 3300–3650 cm^−1^, which widened and moved towards lower wavenumbers owing to the stretching vibration of the hydroxyl groups—that is, the hydrogen bonding between PVA and BC. The peak tensile vibration of methylene C-H was observed at approximately 2930 cm^−1^. This indicated that there are many -OH and -CH_2_ groups in the PBC hydrogel. The peak value at approximately 1030 cm^−1^ indicates the stretching vibration of the -COO bond in the C-O-C group, whereas the absorption peak values at 1707 and 1745 cm^−1^ indicate the stretching vibration of the -COO bond in CA [26]. At 1700 cm^−1^, the tensile damping peak of -COOH disappeared with the introduction of PVA and BC, whereas the in-plane bending damping peak of the C=O bond appeared at 1600 cm^−1^, indicating that the carboxyl group on CA reacted with the hydroxyl group on the PB hydrogel.

### 2.3. X-Ray Diffraction Analysis of the Hydrogels

**Figure 3:**
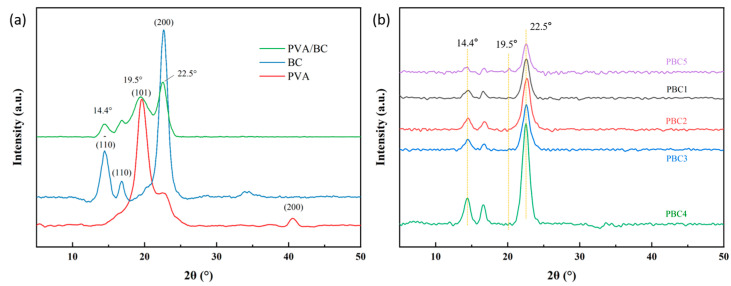
(**a**) XRD patterns of original BC, PVA, and PVA/BC hydrogel samples. (**b**) XRD patterns of PBC samples with different PVA contents.

The XRD pattern of the original PVA showed intense peaks located at 2θ = 19.8° (101) and 2θ = 40.6° (200°). BC exhibited three diffraction peaks at 14.4°, 16.8°, and 22.5°, corresponding to the (110), (110), and (200) crystal planes of BC, belonging to the crystal structure of cellulose Iα. The XRD peaks of the PVA-BC hydrogel at 14.4°, 19.5°, and 22.5° correspond to the crystal structure of cellulose I [27]. The complexation and salting-out of PVA-based hydrogels with salts have been extensively studied. Figure 3 shows the XRD patterns of the PBC hydrogels. The main peak of the hydrogels is also located at 2θ = 22.5°, and with the increase in PVA content, the intensity of the main peak of BC increases. This indicates that the crystallinity of the hydrogels increased, which may be due to the entanglement of BC and PVA chains and the enhancement of the interaction between BC and PVA molecules.

When the PVA concentration was 5% (PBC5 samples), the 2θ = 22.5° and 14.4° diffraction peaks of the hydrogel were less intense, whereas the intensity of the diffraction peak 2θ = 19.5° was enhanced. This may be attributed to the high concentration of PVA and the role of salt in the immersion process, which promotes the dissociation and chain entanglement of the hydrogel crystallization. PVA tends to form intramolecular hydrogen bonds, which increases its crystallinity through physical crosslinking during freezing and thawing cycles and the formation of microcrystals through the dissolution and rearrangement of these chains, leading to the folding of PVA chains.

The XRD profile presents a rather interesting pattern at low PVA concentrations, where there is a greater tendency to connect with cellulose chains rather than forming intramolecular hydrogen bonds, whereas at high PVA concentrations, more hydrogen bonds are formed within PVA–PVA interactions. In addition, owing to the high orientation of these chains towards the cellulose fibers, they may increase the crystallinity [28].

### 2.4. Morphology Characterization

**Figure 4:**
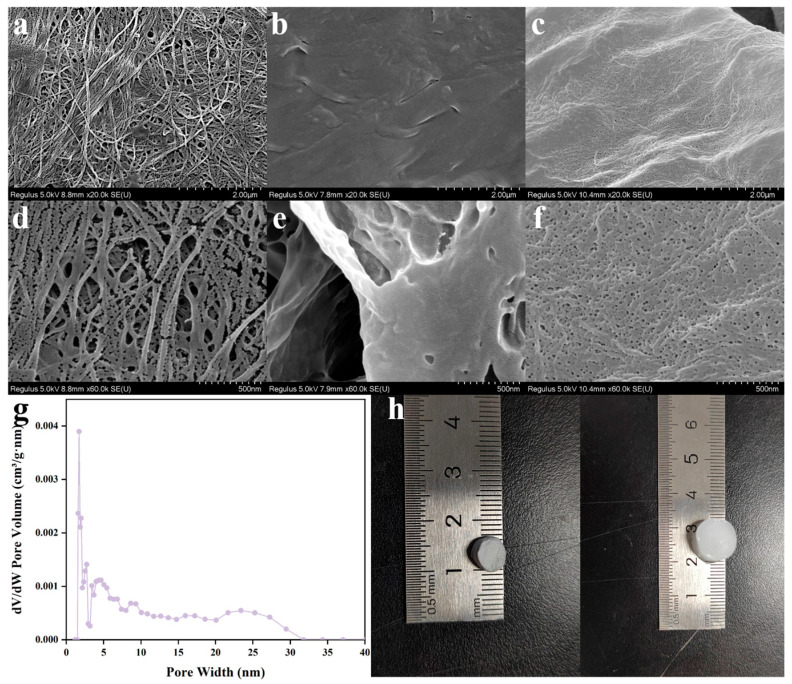
Scanning electron micrographs of 0.8% BC (**a**,**d**), PB (**b**,**e**), and PBC4 (**c**,**f**) at different magnifications. (**g**) Pore distribution diagram of PBC4 hydrogel. (**h**) Digital images of AHB gel dressings before and after lyophilization.

The dense pore structure of the hydrogels effectively promoted the absorption of wound exudates. The micromorphologies of the BC, PB, and PBC4 hydrogels were observed using SEM at two different magnifications (Figure 4). The mass fraction of pure BC in the image is 0.8%, and the mass fraction of PVA in the PB hydrogel is 4%. Compared with pure BC and PB gels, the PBC4 hydrogel showed a porous network structure after immersion in CA and CAT solutions. When the PB aqueous dispersion was frozen, it caused the aggregation of nanocellulose and polymers between ice crystals, resulting in PVA wrapping on the surface of the nanofibers [29]. When heated and crosslinked with CA, a stable, porous nanofiber structure was generated. This is due to the formation of bubbles within the PB hydrogel structure during the crosslinking reaction using the catalyst. This compound releases a large amount of gas during its reaction with the acid, and due to thermal decomposition, additional space and small network structures are formed in the sample.

### 2.5. Gel Fraction Analysis

**Figure 5:**
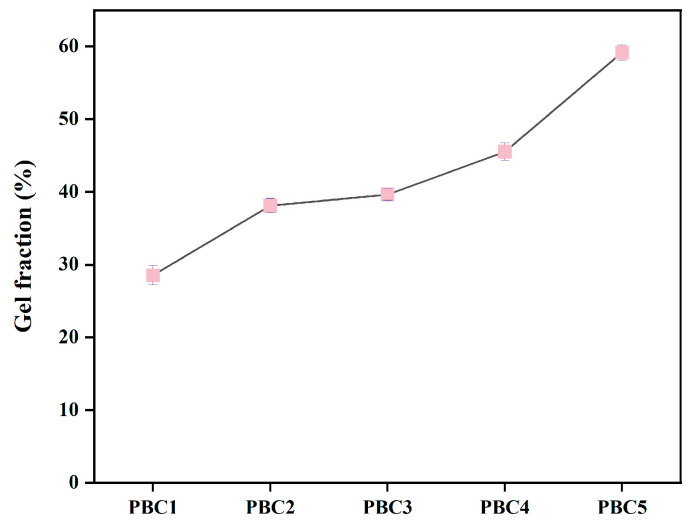
Gel fraction percentage of different hydrogels.

The PBC composite hydrogels were analyzed using the sol–gel method (Figure 5). As the PVA content of the hydrogels increased, an increase in the gel fraction percentage (GF) was observed. The GF of PBC5 was 59%, which was relatively high. The GF value of the hydrogels decreased with decreasing PVA concentration. The GF of PBC1 was 28%, and approximately 70% of the PVA was non-crosslinked; therefore, it could have dissolved in deionized water during the gel composition test. The results show that the degree of crosslinking of the polymer network in the hydrogel was strengthened by increasing the PVA content, and the crosslinking reaction of the BC-PVA hydrogel mainly occurred on the surface of the CA–CAT solution.

### 2.6. Water Vapor Transmission Rate (WVTR)

**Figure 6:**
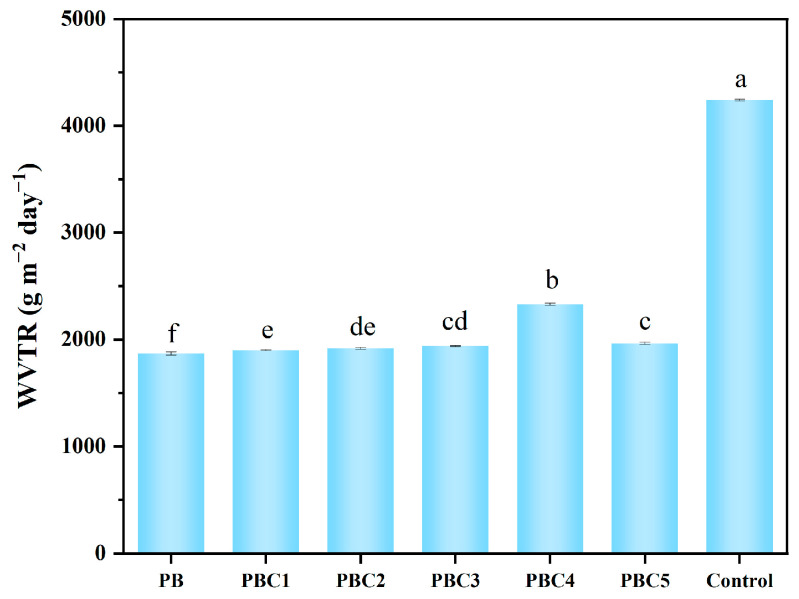
Water vapor transmission rates. Letters above the bars indicate significant differences in the values of different hydrogels at *p* < 0.001.

Water vapor permeability is a key parameter for testing the suitability of hydrogels for wound dressings. Moisture permeability in hydrogels prevents the accumulation of exudates in the wound, thus accelerating wound healing. However, excessive water loss can lead to wound dryness, thereby delaying healing. For wound dressings, the water vapor permeability should be maintained at an ideal level—excessive levels can lead to extreme water loss, and extremely low levels may result in exudate leakage. Therefore, the WVTR is a key parameter that determines the suitability of hydrogels for wound dressing. As shown in Figure 6, the WVTR values for PB, PBC1, PBC2, PBC3, PBC4, and PBC5 were 1872 ± 16, 1902 ± 4, 1919 ± 10, 1940 ± 6, 2332 ± 9, and 1967 ± 11 g m^−2^ day^−1^, respectively. These values indicate the medium permeability of the hydrogels, which was within an acceptable range. However, the WVTR of the control group (without coverage) was 4242 ± 8 g m^−2^ day^−1^, which was very high.

### 2.7. Swelling Capacity of Hydrogel

**Figure 7:**
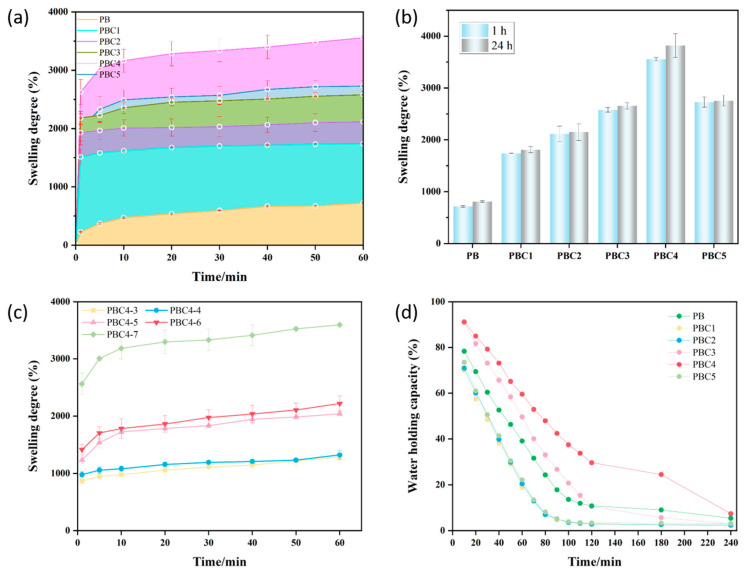
Water-related properties of hydrogels. (**a**) Swelling rates (%) of hydrogels cultured in water for more than 60 min. (**b**) Swelling rates (%) of the hydrogel samples incubated in water for 60 min compared with those of the hydrogel samples incubated in water for 24 h. (**c**) Swelling rates (%) of hydrogels under different freeze–thaw cycles, cultured in water for more than 60 min. (**d**) Water holding capacity of different kinds of hydrogels at 37 °C (%).

The absorption of exudates is a key aspect in moist wound care. Wound dressings that maintain a moist environment on the surface of the wound promote the migration and epithelialization of wound cells, which prevents excessive saturation of exudates from damaging the surrounding skin [30]. For a dressing, it is critical that the hydrogel absorbs a large amount of wound exudate without losing its state and without the dressing adhering to the wound bed. This property accelerates the transport of nutrients and cell products between the hydrogel and the surrounding media of the wound. The liquid absorption characteristics of the prepared hydrogels are shown in Figure 7a. When the hydrogels were incubated with water for more than 60 min, the swelling rate of all hydrogel samples increased significantly with time. At 60 min, the PBC4 hydrogel exhibited the highest swelling rate (3485.3%). This is because the nanopores increased the specific surface area of the hydrogel, thus promoting water absorption. A higher PVA content resulted in a significantly lower swelling rate. On the contrary, a lower PVA content reduced the crosslinking density and degree of entanglement, making it easier for the PBS solution to penetrate the hydrogel, which increased the degree of swelling. Therefore, the higher the PVA content is, the lower the porosity is. Furthermore, the smaller is the pore diameter is, the smaller the amount of PBS solution entering the hydrogel structure is. By comparing the swelling rate for different freeze–thaw cycles, the corresponding conclusion was also confirmed (Figure 7c). Therefore, compared with other hydrogels, the PBC4 hydrogel showed the highest swelling rate, mainly due to its hydrophilic groups and porous surface structure.

Except for PBC4, all the other samples reached equilibrium after soaking for 1 h (Figure 7b). Compared to other groups, the PBC4 samples showed higher swelling rates after soaking for 1 h and 24 h. We suspect that this may be due to the higher PVA content, which led to significantly stronger crosslinking of the polymer network of PVA and BC in the hydrogel, a weakened esterification reaction with CA, and an increase in the free -COOH group of CA in the hydrogel. When the pH of the swelling medium exceeded the constant value for CA dissociation in the hydrogel, the free -COOH group of CA in the hydrogel gradually ionized into -COO-, and the net surface charge on the hydrogel became negative. After the gel was ionized, an increase in the electrostatic repulsion between the negatively charged carboxylate groups led to an increase in the swelling of the hydrogel network, which improved the porosity of the gel.

The expansion process of these hydrogels better fits Schott’s second-order kinetic equation model (Table 1), which describes the swelling process caused by the diffusion of hydrogels in an aqueous solution during hydration. The values in Table 1 agree with the model and completely predict the absorption capacity and swelling rate of all hydrogels studied. Thus, an increase in PVA content increases the swelling rate. Meanwhile, an excessive PVA content leads to a decrease in the swelling rate, which may be attributed to the decrease in the affinity of the hydrogel network for water molecules, making it more difficult for water to diffuse into the sample.

The theoretical values and the maximum water absorption values from the experiment are similar, but there are differences in the behavior of the expansion rate depending on the PVA content.

### 2.8. Water Holding Capacity

The water retention capacity of the PBC hydrogels decreased over time (Figure 7d). Compared with other gels, the PBC4 hydrogel showed a higher water holding capacity. The PBC1, PBC2, and PBC5 hydrogels lost almost all the water within 100 min, whereas PBC4 retained almost 30% of the initial water volume and also retained some water after 240 min. In general, PBC hydrogels have a porous surface structure, high swelling capacity, and good water retention capacity, which helps to absorb exudates and keep the wound surface wet, making them a promising material for wound dressings (Table 2).

### 2.9. Cytotoxicity Assessment by CCK-8

**Figure 8:**
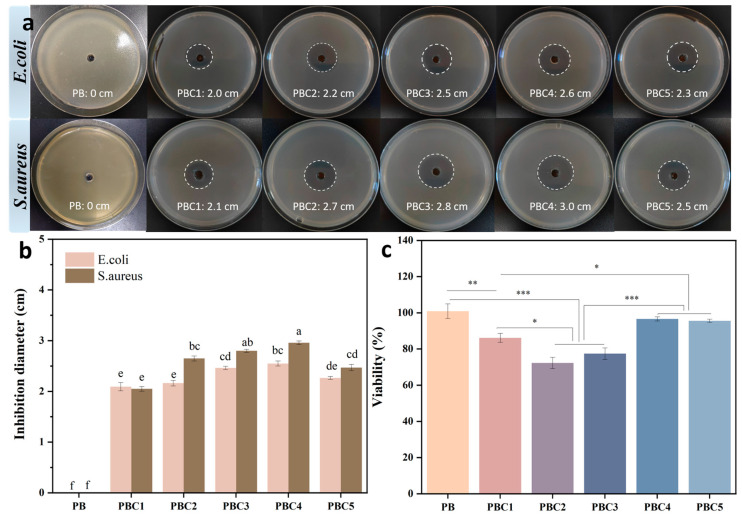
Study on antibacterial activity. (**a**) Photos of inhibition zones of different kinds of hydrogels against *Escherichia coli* and *Staphylococcus aureus*. (**b**) Diameter of inhibition ring of different hydrogels; different letters indicate significant differences in values (*p* < 0.001, n = 3). (**c**) Cell viability (%) of PBC1, PBC2, PBC3, PBC4, and PBC5 hydrogels, determined by CCK-8 assay on L929 normal fibroblast cell line (n = 3; * *p* < 0.05, ** *p* < 0.01, *** *p* < 0.001).

Due to the physical contact between a hydrogel dressing and wound tissue, hydrogels used as a wound dressing must be biocompatible [36]. The cytocompatibility of all the hydrogel samples was evaluated using the CCK-8 method, and the results are plotted in Figure 8c. The results of the cell viability test show that the PBC4 hydrogel had significant cytocompatibility and retained approximately 96.9% cell viability. In contrast, the activity of the hydrogel cells was negatively affected by changing the PVA content. The cell viability of PBC5 was similar to that of PBC4, whereas the cell viability of PBC1, PBC2, and PBC3 decreased to nearly 86%, 71.7%, and 77.4%, respectively.

### 2.10. Antimicrobial Activity

Wound dressings with antibacterial properties can prevent infections and promote wound healing. The antibacterial activities of the different hydrogel samples against Gram-positive bacteria (*S. aureus*) and Gram-negative bacteria (*E. coli*) are shown in Figure 8a. All PBC hydrogel samples showed obvious bacteriostatic circles. Among them, PBC4 had the largest inhibition zone diameters against *S. aureus* and *E. coli* (3.0 cm and 2.6 cm, respectively). All the samples had a significant inhibitory effect on *S. aureus*. This is mainly because of the antibacterial mechanism of organic acid antibacterial agents and the structural differences between Gram-positive and Gram-negative bacteria. Moreover, the antibacterial mechanism by which metal ions (Ag) interact with sulfur-containing proteins in bacterial cell walls, leading to bacterial death, is different [37]. The antibacterial activity of organic acids depends on their pKa value. Therefore, they are more active under acidic conditions, because the proportion of undissociated organic acids remaining in the hydrogel is high, which can pass through the bacterial membrane and decrease the pH of the cell after the dissociation of hydrogen ions [38].

## 3. Materials and Methods

### 3.1. Materials

BC dispersion, 0.8wt% was obtained from Qihong Technology Co., Ltd. (Guilin, China). Disodium hydrogen phosphate and CA were purchased from Sinopharm Chemical Reagent Co., Ltd. (Shanghai, China). Sodium bicarbonate was purchased from the Tianjin Damao Chemical Reagent Factory. PVA (alcoholysis degree: 98–99%) was purchased from Macklin Biochemical Technology Limited (Shanghai, China). The CCK-8 detection kit, Dulbecco’s Modified Eagle Medium (DMEM), and trypsin ethylenediaminetetraacetic acid (trypsin EDTA) digestion solution were purchased from Solarbio Science and Technology Co., Ltd. (Beijing, China). L929 mouse skin fibroblasts were purchased from the Global Center for Biological Resources (ATCC). All the reagents were purchased and used directly without further purification.

### 3.2. Preparation of Hydrogels

A certain amount of PVA was dissolved in water at 90 °C for 4 h to obtain a PVA solution. The mixture was combined with a BC (0.55M) suspension under stirring to obtain a uniformly mixed solution. The solutions were mixed, poured into a mold, and frozen at −18 °C. The mixture was frozen for 8 h and then melted for 3 h at room temperature to obtain the PB hydrogel; the freezing and thawing process was repeated seven times.

A 20% CA solution and a catalyst solution containing disodium phosphate and sodium bicarbonate (1:1 mass ratio) were prepared. The hydrogel was then immersed in CA and CAT for 24 h and subsequently thermally crosslinked at 165 °C for 1 h [39,40]. After completion, it was purified with distilled water for 24 h until the pH stabilized at 7.0 to remove the unbound CAT molecules and obtain the PBC hydrogel. Hydrogels with different PVA contents (1%, 2%, 3%, 4%, and 5%) were prepared in the same way and named PBC1, PBC2, PBC3, PBC4, and PBC5. In addition, PBC4-X hydrogels (X: 3–7, representing different freeze–thaw times) were prepared, which were PBC4 hydrogels with different freeze–thaw times. All the hydrogels were prepared under different conditions using the same process.

### 3.3. Characterization of Hydrogels

The chemical compositions of the hydrogel composites were evaluated by Fourier transform infrared spectroscopy (FT-IR) in the wave number range of 500–4000 cm^−1^ using an ALPHA Brook instrument (Dresden, Germany). The crystal structures of the raw materials and hydrogel samples were examined using X-ray diffraction (XRD) with a SmartLab SE instrument (Tokyo, Japan). The microstructure of the hydrogel was observed under a scanning electron microscope (SEM, Hitachi Regulus 8220, Tokyo, Japan). The freeze-dried hydrogels were cut into thin pieces and attached to an SEM bracket with a conductive adhesive. After coating a layer of gold onto the freeze-dried hydrogel samples, the hydrogel morphology was studied.

### 3.4. Gel Fraction

The following steps were performed to determine the gel fraction of the hydrogels. First, the hydrogels were cut into a cylinder and freeze-dried for 12 h (W_0_) using a freeze-drying technology. The dried samples were then immersed in deionized water for 24 h until they reached a constant weight to separate the soluble part of the samples from the solution. Subsequently, the samples were freeze-dried for 12 h (W_g_). The calculation equation for the gel fraction (GF) percentage is as follows:

(1) $$GF\left(\%\right)=\left(\frac{W_{g}}{W_{0}}\right)\times 100$$

where W_0_ is the dry weight of the sample before soaking in deionized water, and W_g_ is the dry weight of the same sample after soaking in deionized water for 24 h.

### 3.5. Swelling Capacity

The hydrogels were dried to completely remove moisture and then weighed. Subsequently, the dry hydrogels (W_d_) were immersed in PBS solution with a physiological pH of 7.4 at 37 °C. The weight of the hydrated hydrogels (W_s_) was measured every 10 min after immersion for 1 min for a total of 60 min. After 60 min, the hydrogel samples were left in water for 24 h and then weighed. The degree of hydrogel swelling was calculated using the following formula:

(2) $$degree\;of\;swelling\left(\%\right)=\left[\frac{{(W}_{s}-W_{d})}{W_{d}}\right]\times 100\%$$

where W_s_ and W_d_ are the swelling weight of the sample at immersion time (t) and the dry hydrogel weight at time (0), respectively.

Next, the effects of PVA concentration and dosage on the properties of the hydrogels were studied. The kinetic model of the second-order equation proposed by Schott (1992) was used to analyze the experimental swelling data:

(3) $$\frac{t}{W}=A+Bt$$

where A is the reciprocal of the initial expansion rate of the hydrogel (A = 1/(dWt/dt); B is the reciprocal of the expansion ratio of the hydrogel under the theoretical equilibrium condition (B = 1/W∞); and Wt and W ∞ are the water absorption capacity of the sample at time t and equilibrium, respectively. A relationship graph was plotted between t/W and t, and the values of W∞ and Ks were calculated using linear regression coefficients; here, Ks is the swelling rate constant (g/g min).

### 3.6. Water Holding Capacity Test

Each dry hydrogel sample was weighed, immersed in deionized water for 24 h to obtain the maximum absorption balance, and then weighed again. The expanded hydrogel was drained with filter paper to remove the surface moisture and placed on a culture dish, which was then placed in an incubator at 37 °C and 40% relative humidity. It was weighed weigh every 10 min for 240 min. The water holding capacity (WHC (%)) was calculated using the following formula:

(4) $$WHC\left(\%\right)=\frac{W_{t}}{W_{0}}\times 100$$

where W_t_ is the residual weight of the water gel at time t during drying, and W_0_ is the initial weight of the water gel at equilibrium.

### 3.7. Water Vapor Transmission Rate

The hydrogel (15.1 mm in diameter) was installed on the edge of a non-corrosive, airtight, and impermeable vial. The vial was filled with deionized water and its opening was covered with polytetrafluoroethylene tape while maintaining a certain gap between the sample and water. The total weight of the measuring device (vial + hydrogel sample + water + polytetrafluoroethylene tape) was determined. Subsequently, the entire device was placed in an environment at 37 °C and 84% humidity for 24 h. In addition, we used the same type of vials with no hydrogel samples and polytetrafluoroethylene covers as negative controls. After 24 h, the equipment was removed and reweighed. The water vapor transmittance rate was calculated as follows:

(5) $$WVTR=((W_{2}-W_{1})/A)\times 24$$

where W_1_ and W_2_ are the initial and final total weights, respectively, and A is the area of the hydrogel sample used for penetration.

### 3.8. Cytocompatibility Analysis

#### 3.8.1. Cell Culture

The L929 (mouse fibroblast) cell line was cultured in a modified complete DMEM containing 10% FBS, 2 mM glutamic acid, 4.5 g/L glucose, 1% penicillin, and 1% streptomycin (100 μg/mL) and placed in a saturated humidity environment at 37 °C and 5% CO_2_ to evaluate the biocompatibility of the PBC hydrogel samples. The cells were separated using 0.25% trypsin diluted in PBS and then arranged into a single cell suspension of 1 × 10^4^/mL in a new culture medium for cell viability experiments.

#### 3.8.2. Cell Toxicity Assessment

The cell activity of the hydrogels was determined using the CCK-8 method. Each hydrogel was sterilized at 50 % relative humidity for 2 h and then incubated in DMEM at 37 °C for 3 d. An extract was obtained by passing the hydrogel-containing medium through a filter. Next, 200 μL of the L929 cell suspension (1 × 10^4^ cells/well) was added and inoculated into a 96-well plate and then cultured for 24 h at 37 °C and 5% CO_2_. Subsequently, the culture medium was removed and 200 μL of fresh culture medium (containing 50 μL of extract) was added. A culture medium without the extract was used as the control. After incubating at 37 °C for 24 h, equal amounts of CCK-8 were added to each well and further incubated at 37 °C for 90 min. The OD was recorded at 450 nm using an enzyme-linked immunosorbent assay (HM-SY96A).

### 3.9. In Vitro Antibacterial Performance

One milliliter of a suspension of *Escherichia coli* (*E. coli*) or *Staphylococcus aureus* (*S. aureus*) at a concentration of 1 × 10^8^ CFU/mL and 10 mL of LB agar broth were poured into a culture dish. Each sterile hydrogel was then placed in a bacterial agar plate with a pore size of 6 mm^−1^ and incubated at 37 °C for 24 h [41,42]. The suppression areas of the measurements were recorded.

### 3.10. Statistical Analysis

All experimental results are presented as the mean ± standard deviation (SD). Statistical processing was performed using ANOVA with Scheffé’s post hoc test. A value of *p* < 0.05 was considered to indicate a significant difference.

## 4. Conclusions

In this study, a hydrogel-based wound dressing was developed for high absorption of wound exudates using BC, PVA, and CA through a simple and feasible preparation method. The surface porosity, water absorption, and water retention of the hydrogels were adjusted by changing the PVA content. The newly prepared hydrogel material demonstrated quick water absorption and slow release of the absorbed water. In addition, the CCK-8 assay results showed that the prepared PBC hydrogel was biocompatible and had excellent antibacterial properties. The developed hydrogel not only absorbs excess exudates from wounds but also maintains a moist environment for dry wounds, providing optimal environmental conditions for promoting cell attachment and migration. These characteristics make the developed samples highly promising candidates for use as wound dressings.

## Figures and Tables

**Table 1:** Theoretical correction value of obtained hydrogels using Schott’s equation.

Sample	R^2^	Ks	W_∞_calc	W_∞_obs
PBC1	0.9999	0.0573	17.44	18.42
PBC2	0.9998	0.0443	22.57	22.67
PBC3	0.9999	0.0386	25.90	27.24
PBC4	0.9990	0.0283	35.31	40.82
PBC5	0.9997	0.0347	28.80	28.39

Ks: swelling rate of hydrogel; W∞ calc: theoretical value of hydrogel swelling; W∞ obs: experimental value.

**Table 2:** Comparison of the water absorption and water vapor permeability performance with previous reports.

Material	Swelling Rate at 24 h (%)	Water Vapor Transmission Rate (g m^−2^ day^−1^)	Reference
PVA/SPI	838.8	2430.8	[31]
PU/PAN-SPA	950	2200	[32]
GA/AgNPs/PVA/PCL	540.53	2193.27	[33]
CA/Gel/CS	450	3419	[34]
PVA/CS/nZnO	9.3	2200	[35]
PVA/BC/CA	3485.3	2332.1	This work

## Data Availability

All data generated or analyzed during this study are included in this published article.
